# Experimental glass ionomer cement modified by the incorporation of nanoselenuim: antibacterial activity, cytocompatibility and compressive strength

**DOI:** 10.1186/s12903-025-07504-y

**Published:** 2025-12-27

**Authors:** Mariam Romany Wadee, Yasser F. Gomaa, Reem Gamal

**Affiliations:** 1 Biomaterials Department, Faculty of Dentistry, Assiut University, Al-Gamaa st., Naila Khatoon, Assuit, 71517 Egypt; 2 Biomaterials Department, Faculty of Dentistry, Minia University, Minia, 61519 Egypt

**Keywords:** Atraumatic restorative treatment (ART), Minimum inhibitory concentration (MIC), Selenium nanoparticles, Streptococcus mutans (S. mutans), Glass ionomer cement (GIC), Sulforhodamine B (SRB) assay, Cytotoxicity

## Abstract

**Background:**

The use of restorations with antibacterial activity has become mandatory to control secondary caries, especially in atraumatic restorative treatment (ART). Therefore, this study was conducted to enhance the antibacterial activity of conventional glass ionomer cement (GIC) by adding nanoselenium (NSe) and to assess the impact on its cytocompatibility and compressive strength.

**Methods:**

NSe was prepared and characterized, and its minimum inhibitory concentration (MIC) against *Streptococcus mutans (S. mutans)* was determined via the broth microdilution technique. Based on the MIC, grouping was performed. Group I included unmodified GIC samples mixed with water, and groups II–IV included GIC samples mixed with three different concentrations (75, 112.5, and 150 ppm, respectively) of the NSe suspension. Antibacterial activity against *S. mutans* was assessed via an agar disc diffusion test over four time intervals (24, 48, 72 h, and 7 days). The cytocompatibility of 100% and 10% concentrations of the sample extract was evaluated via a sulforhodamine B (SRB) assay against oral epithelial cells. Additionally, compressive strength testing was performed according to ISO 9917-1 using a universal testing machine.

**Results:**

Regarding antibacterial activity, group IV presented significantly higher values than the other groups, followed by group III, at all time intervals. For cytocompatibility, group II had higher values of cell viability at both concentrations. For all groups, the 10% concentration had significantly higher values of cell viability than the 100% concentration. Moreover, none of the groups showed a statistically significant difference in compressive strength.

**Conclusions:**

The addition of NSe at concentrations up to 150 ppm resulted in extended antibacterial activity against *S. mutans* for up to 7 days without affecting its compressive strength. Furthermore, the addition of NSe at a low concentration, such as 75 ppm, increased the viability of oral epithelial cells.

## Background

Glass ionomer cement (GIC) is an acid-based cement formed by mixing fluorocalcium aluminosilicate glass powder with a polyacrylic acid solution [1–5]. Since its development, it has been employed in a variety of dental applications, such as direct filling, base material, luting cement, and pit and fissure sealant [1, 5–7]. This is attributed to its unique properties, including chemical adherence to the tooth structure, fluoride release potential, a thermal expansion coefficient matched with that of hard dental tissues, acceptable esthetics, and optimal biocompatibility [1–9]. These characteristics make GIC a preferable material for atraumatic restorative treatment (ART), patients with a high caries index, and pediatric dentistry [1, 3–6]. ART is a minimal intervention approach based on the removal of demineralized tooth tissues using hand instruments. In this technique, the cavity must be restored with adhesive restorative materials. Compared with rotary burs, hand instruments may be unable to efficiently remove carious dentin. Hence, clinical success depends on the ability of the restorative material to arrest the carious process [1, 3, 6]. The antibacterial potential of GIC depends mainly on its initial low pH during the setting reaction [8] and its fluoride release ability. Studies have reported that the common reason for GIC filling failure is the development of secondary caries, indicating that the amount of fluoride released from traditional GIC is inadequate to fulfill the intended antimicrobial effects [1, 4, 7–9]. Therefore, the modification of GIC is needed to increase its anticariogenic ability and achieve better clinical outcomes when different antimicrobial agents are added.

Selenium (Se) is an essential trace element for diet maintenance and is present in 100 selenoproteins. It has numerous health benefits, such as anticancer activities, and plays an important role in the immune system [10–14]. It serves as the basis for selenoenzymes such as glutathione peroxidase, an antioxidant that scavenges several types of free radicals and reactive oxygen species (ROS) to protect tissues [11–14]. Se deficiency has been associated with increases in both viral and bacterial infections, as well as several systemic diseases [13–15]. However, the safety margin of Se is very narrow, so it has limited therapeutic use [14, 16], with a tolerable upper intake level (UL) of 400 µg/day [17].

Biological and biomedical science has advanced enormously with the aid of nanotechnology [4]. Nanoparticles (NPs) display great potential as antimicrobial agents [7, 9, 10, 18, 19]. Among their distinctive characteristics are their small size, large surface area-to-volume ratio [7, 9–11], high surface energy, and spatial confinement [10], with a minimal risk of developing microbial resistance [19]. Compared with macro-Se, nanoselenium (NSe) has drawn great attention because of its biocompatibility, chemical stability, low toxicity, and greater effectiveness [10, 11, 13–15, 20, 21]. Previous studies have shown that NSe has anticancer, antioxidant, anti-inflammatory, and immune-stimulating activities. Furthermore, NSe has been reported to be effective against many pathogens, including *Streptococcus mutans (S. mutans)*, *Enterococcus faecalis*, *Staphylococcus aureus*, *Escherichia coli*, and *Candida albicans* [10, 11, 13–15, 19, 22].

Synthesis of NSe can be performed chemically, physically, or biologically [10, 11, 14, 15, 19, 22]. Chemical synthesis usually involves the reduction of Se precursors by reducing agents, physicochemical reduction, or electrochemical techniques [10, 11, 15, 16], followed by stabilization with different materials, such as polysaccharides, proteins, and surfactants [11, 14–16]. Stabilization of NSe is necessary because NSe in the zero oxidation state is highly unstable and can be transformed into a nonbioactive gray analog [16, 21]. Meanwhile, physical synthesis is carried out through excitation and the release of electrons via energy, which results in the reduction of high-valence Se sources into stable elemental Se [14]. Physical methods have multiple drawbacks: they are complicated, difficult, and require specific equipment [13, 15]. Moreover, the temperature must be carefully controlled, as it strongly affects the morphology and size of NSe [23].

On the other hand, biological (green) synthesis can be distinguished into biogenic synthesis, using cellular structures such as bacterial cultures, fungi, or yeasts, and bioorganic synthesis, using noncellular extracts from various plants to synthesize NSe [11, 14, 15, 19, 22]. As a drawback, in biogenic green synthesis, NSe can be capped with proteins, enzymes, or some cellular residues, which are almost impossible to separate and interfere with NSe applications [13, 14, 24]. Meanwhile, in green bioorganic synthesis, a large volume of plant extract is usually needed, leading to the addition of extra steps, which can be controlled only to a small extent [13, 14].

To date, no study has exploited NSe as an antibacterial additive for GIC. Unfortunately, the inclusion of any antibacterial agent in GIC results in alterations to its characteristics, particularly its cytotoxicity and compressive strength. For these reasons, this study sought to modify GIC with varying NSe concentrations to assess their impact on antibacterial activity, cytocompatibility, and compressive strength. The null hypothesis of this study was that the incorporation of NSe into GIC would not significantly affect its antibacterial activity, cytocompatibility, or compressive strength.

## Materials and methods

### Preparation of NSe suspension

NSe water-based suspensions were obtained, sterilized, and characterized by Nano Gate Co. (Mokatam, Cairo, Egypt). They were prepared as described by Zhang et al. [16] through the chemical reduction of sodium selenite (Chem-Lab, Belgium) by ascorbic acid (Loba-Chemie, India), using chitosan (Cts) as a stabilizing agent (medium molecular weight, DD = 85%, Loba-Chemie, India), which is the only positively charged natural polysaccharide with good solubility in acetic acid and excellent biocompatibility [16]. This method provides high purity and enables the use of precise concentrations with effective control over mass production [15].

One gram of Cts and 0.8 g of ascorbic acid were completely dissolved in 100 mL of 1% (w/w) acetic acid (Millipore Merck, India) to obtain a Cts/ascorbic acid solution. Then, 5 mL of an aqueous sodium selenite (Na_2_SeO_3_) solution containing 0.2 g of sodium selenite was added dropwise to the Cts/ascorbic acid solution and stirred vigorously (Cimarec+™ Stirring Hotplate, Thermo Scientific, USA) at 500–600 rpm for 15 min. After preparation, the suspension was sterilized using a syringe filter.

### Characterization of the NSe suspension

Characterization was performed via transmission electron microscopy (TEM) with high resolution (JEM-2100 F, JEOL Ltd., Japan) at an accelerating voltage of 200 kV. The samples for TEM were prepared by placing a droplet of colloid suspension in the relevant solvent onto a Formvar carbon-coated, 300-mesh copper grid (Ted Pella) and allowing it to evaporate in air under ambient conditions. Moreover, the prepared particles were analyzed for their particle size and size distribution in terms of the average volume diameters and polydispersity index using photon correlation spectroscopy with a particle size analyzer via dynamic light scattering (DLS) (Zetasizer Nano ZN, Malvern Panalytical Ltd, UK) at a fixed angle of 173° and a temperature of 25 °C. Samples were analyzed in triplicate. The same equipment was used for the determination of zeta potential (ZP), which assesses the surface charge of NPs. This is important for evaluating the electrostatic repulsive forces between charged particles in the colloid state and the affinity of NPs for bacterial cell membranes.

### Minimal Inhibitory Concentration (MIC) of the NSe suspension

The MIC was measured according to the Clinical Laboratory Standards Institute (CLSI) standard protocol [25] via the broth microdilution technique in a 96-well microplate against the ATCC 25175 type *S. mutans* strain obtained from the Microbiological Resources Centre (MIRCEN, Cairo, Egypt). All culture media and equipment used for the assays in this study were obtained pre-sterilized or autoclaved according to standard laboratory protocols. One hundred microliters of bacteria were cultured in brain heart infusion (BHI) broth. The culture was diluted to reach 0.5 McFarland turbidity standards (BIOMERIEUX SA., France) and then pipetted into the wells. This was followed by the addition of 100 µL of 5 serial dilutions (100, 50, 25, 12.5, and 6.25%) of NSe suspension, where 100% was equivalent to 150 ppm. A negative blank control was used. Additionally, a positive control was prepared using 10 µg/mL amoxicillin. After 48 h of incubation, a microplate reader (Stat Fax 2100, Awareness Technology, Inc., Palm City, USA) was used to detect the optical density at 600 nm.

### Sample size calculation and study design

The sample size calculation was executed via G*Power version 3.1.9.7 [26] by adopting an (α) level of 0.05 and a (β) of 0.2 (i.e., power = 80%). For antibacterial activity and compressive strength, effect sizes (f) of 0.753 and 0.677, respectively, were calculated based on the results, according to Bayoumi and Habib [27]. The sample size was determined to be a total of 24 samples for antibacterial activity (*n* = 6/group), while for compressive strength, it was determined to be a total of 28 samples (*n* = 7/group). Furthermore, according to the ISO 10993-5 [28] standard for the biological evaluation of medical devices—Tests for in vitro cytotoxicity, the sample size for the cytocompatibility test was 12 samples (*n* = 3/group).

A total of 64 water-settable GIC (Kromoglass 2, LASCOD, Florence, Italy) (Lot no. 0472340103) samples were used. Grouping was performed according to the NSe concentration with respect to the MIC against *S. mutans*. The samples were divided into four groups (I–IV): Group I: unmodified GIC, in which the liquid was distilled water. Groups II–IV were prepared by blending the powder with 75, 112.5, and 150 ppm of NSe suspensions, respectively. These concentrations are double, triple, and quadruple the obtained MIC value (37.5 ppm) in the same order.

### Sample preparation

Disc-split Teflon molds (5.7 ± 0.1 mm diameter × 2 ± 0.1 mm height) were used to prepare the samples under aseptic conditions for antibacterial activity and cytocompatibility tests. For the compressive strength test, cylindrical split Teflon molds (3 mm diameter × 6 mm height) were used to prepare the samples according to ISO 9917-1 [29].

The molds were placed on a glass slab coated with insulating material. The samples used in this study were prepared according to the manufacturer’s instructions at a 5:1 g P/L ratio. The mixture was injected into the molds using a plastic syringe. A 500 g weight was then placed on another glass slab, which was positioned over the molds. After setting, the samples were removed from the molds to proceed with the procedures of each test. To ensure blinding and randomization, each group of samples was assigned a different code (name) by an independent coworker, which remained concealed until the day of results interpretation.

### Antibacterial activity test

Antibacterial activity was evaluated via an agar disc diffusion test. The samples were placed immediately after preparation onto BHI agar plates inoculated with a 0.5 McFarland turbidity standard of *S. mutans* ATCC 25175 inoculum. Each agar plate contained one sample from each group, with a total of four samples per plate. The agar plates were subsequently placed into a candle jar and incubated (Precision incubator, Thermo Fisher Scientific, Ohio, USA) at 37 °C for 7 days. The diameters of the inhibition zones (IZO) produced around the samples were measured using a digital caliper in mm at 24, 48, 72 h, and 7 days of incubation. The diameter of the IZO was measured at three different points to obtain the mean IZO for each sample.

### Cytocompatibility test

Cytocompatibility against oral epithelial cells was evaluated via a sulforhodamine B (SRB) assay described by Pinto et al. [30]. The human primary oral epithelial cell line (AcceGen, catalog number ABC-TC4365) was obtained from Nawah Scientific, Inc. (Mokatam, Cairo, Egypt). The cells were maintained in Dulbecco’s modified Eagle’s medium supplemented with 100 mg/mL streptomycin, 100 units/mL penicillin, and 10% heat-inactivated fetal bovine serum in a humidified 5% (v/v) CO₂ atmosphere at 37 °C. The sample extract was prepared according to ISO 10993-5 [28]. Where the prepared samples, after 24 h, were subjected to ultraviolet radiation for sterilization. Next, 1 mL of the cell culture media was added to each sample and incubated at 37 °C for 72 h. Two concentrations of sample extract were used. The nondiluted extract was set as the 100% concentration, while the 10% concentration was obtained by diluting the extract.

Aliquots of 100 µL of oral epithelial cell suspensions (5 × 10³ cells/well) were distributed in a 96-well plate and incubated in complete media at 37 °C for 24 h. The cells were treated with another aliquot of 100 µL of media containing the sample extract. After 72 h, the cells were fixed by replacing the media with 150 µL of 10% trichloroacetic acid and incubated for 1 h at 4 °C. Then, the solution was removed, and the cells were washed five times with distilled water.

Aliquots of 70 µL of SRB solution (0.4% w/v) were added and incubated in the dark at room temperature for 10 min. The plates were washed three times with 1% acetic acid and allowed to air dry overnight. Then, 150 µL of Tris (hydroxymethyl) aminomethane buffer (10 mM) was added to dissolve the protein-bound SRB stain, and the absorbance was measured at 540 nm using an Infinite F50 microplate reader (TECAN, Switzerland).

### Compressive strength test

A total of 28 samples were evaluated according to ISO 9917-1 [29]. After setting, the samples were removed from the molds, finished, and stored at relative humidity for 24 h. A digital caliper was used to check the dimensions of each sample. The samples were vertically positioned on a universal testing machine fixture (Instron 3366, USA). A compressive load was applied parallel to the long axis of each sample at a crosshead speed of 0.5 mm/min until fracture. To calculate the compressive strength in MPa, the maximum force was recorded and divided by the sample cross-sectional area.

### Statistical analysis

Numerical data are presented as means and standard deviations (SD). The normality, variance homogeneity, and sphericity assumptions were verified by examining the data distribution and using Shapiro–Wilk’s, Levene’s, and Mauchly’s tests, respectively. The antibacterial activity data were analyzed via two-way mixed-model ANOVA. The cytocompatibility data were analyzed via two-way ANOVA. Tukey’s post hoc test was performed after one-way ANOVA was used to analyze the compressive strength. In the case of nonsignificant interaction in multilevel ANOVAs, comparisons of main effects were performed via Tukey’s post hoc test for independent variables and Bonferroni post hoc correction for repeated measurements. Simple effect comparisons were performed using the pooled error term from the main ANOVA model, with p values adjusted using Bonferroni correction in the case of a significant interaction. For all tests, the significance level was set at *p* < 0.05. R statistical analysis software, version 4.4.1 for Windows [31], was used to conduct the statistical analysis.

## Results

### Characterization of the NSe suspension

**Fig. 1:**
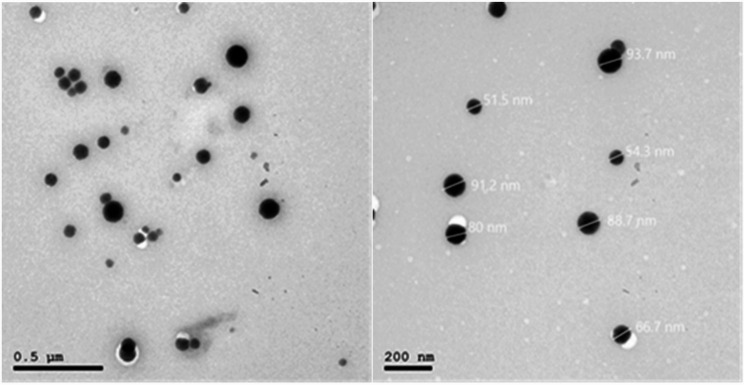
Shows TEM images of NSe particles

TEM analysis revealed NSe particles with a spherical morphology and an average size of 75 ± 5 nm (Fig. 1). The TEM image of the produced NPs shows a high surface area, which allows for a larger contact area between the NPs and the bacterial cell membrane, resulting in cell damage.

The average hydrodynamic diameter of NSe measured by DLS was found to be 134 nm, which is usually larger than that observed by TEM, and the ZP was + 42 mV. This recorded ZP indicates strong electrostatic repulsion between the NPs according to Bhattacharjee [32], who classified NPs dispersions with ZP values of ± 20–30 mV and > ± 30 mV as moderately and highly stable, respectively. A high ZP of NPs prevents their agglomeration in solution and results in a stable suspension.

### Antibacterial activity test

**Fig. 2:**
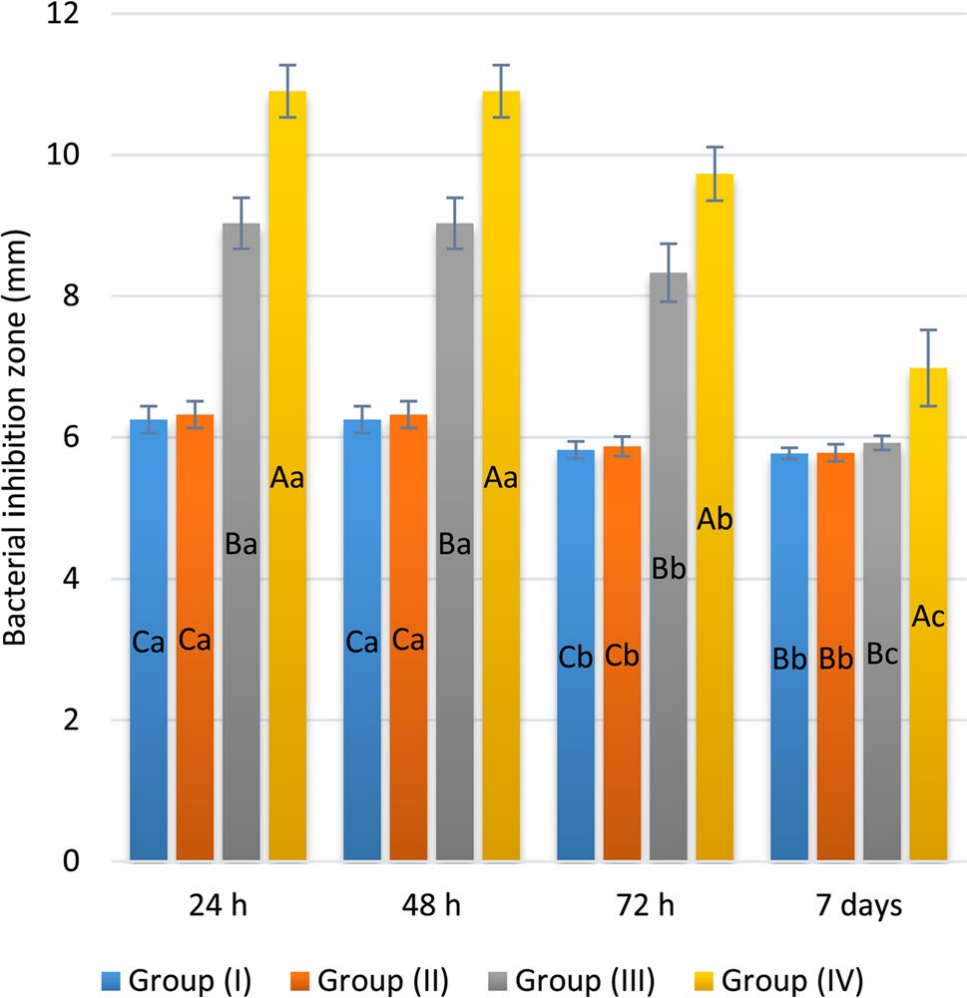
Bar chart displaying the mean and SD values of bacterial IZO (mm). (Different upper superscripts indicate a statistically significant difference within the same interval, and different lower superscripts indicate a statistically significant difference within the same group)

Regarding the antibacterial activity against *S. mutans*, the means and SD of IZO (mm) after 24, 48, 72 h, and 7 days of incubation are shown in Table 1. The differences between the mean values of all groups were statistically significant (*p* < 0.001) (Fig. 2). Group IV had statistically significantly higher mean values at all time intervals (*p* < 0.001). Meanwhile, the mean values of group III were statistically significantly higher than those of groups I and II at the 24, 48, and 72 h time intervals (*p* < 0.001).

**Fig. 3:**
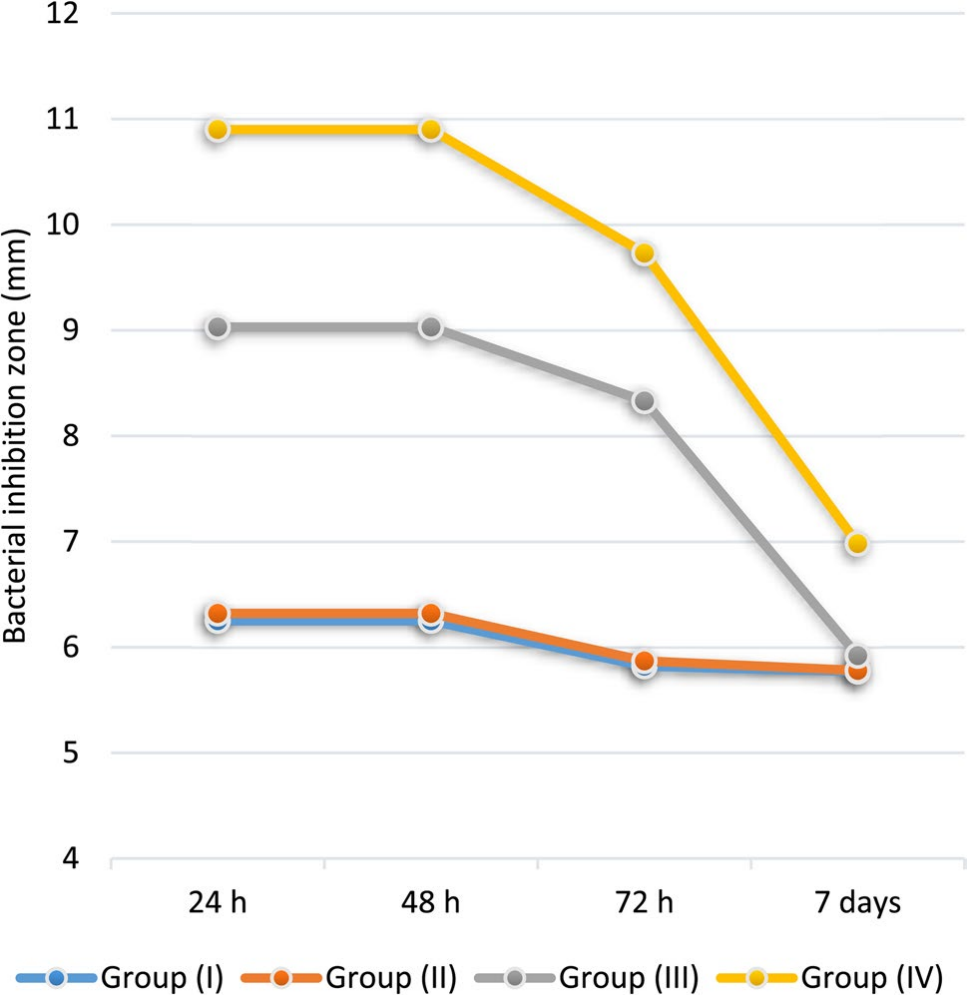
Line chart displaying the mean and SD values of bacterial IZO (mm) over time

A statistically significant difference (*p* < 0.001) was identified between the mean values recorded at different intervals (Fig. 3). The mean values of all the groups were statistically significantly higher at 24 and 48 h than at the other time intervals (*p* < 0.001). In addition, the mean values measured after 72 h were statistically significantly higher than those measured after 7 days for groups III and IV only (*p* < 0.001).

**Table 1:** Simple effects comparisons and summary statistics of bacterial IZO (mm)

Time	Bacterial inhibition zone (mm) (Mean ± SD)				*p*-value
	Group (I)	Group (II)	Group (III)	Group (IV)	
**24 h**	6.25 ± 0.19^Ca^	6.32 ± 0.19^Ca^	9.03 ± 0.36^Ba^	10.90 ± 0.37^Aa^	**< ** **0.001** *
**48 h**	6.25 ± 0.19^Ca^	6.32 ± 0.19^Ca^	9.03 ± 0.36^Ba^	10.90 ± 0.37^Aa^	**< ** **0.001** *
**72 h**	5.82 ± 0.12^Cb^	5.87 ± 0.14^Cb^	8.33 ± 0.41^Bb^	9.73 ± 0.38^Ab^	**< ** **0.001** *
**7 days**	5.77 ± 0.08^Bb^	5.78 ± 0.12^Bb^	5.92 ± 0.10^Bc^	6.98 ± 0.54^Ac^	**< ** **0.001** *
***p*** **-value**	**< ** **0.001** *	**< ** **0.001** *	**< ** **0.001** *	**<** ** 0.001** *	

Values with different uppercase and lowercase superscripts within the same horizontal row and vertical column, respectively, are significantly different

* Significant (*p* < 0.05)

### Cytocompatibility test

**Fig. 4:**
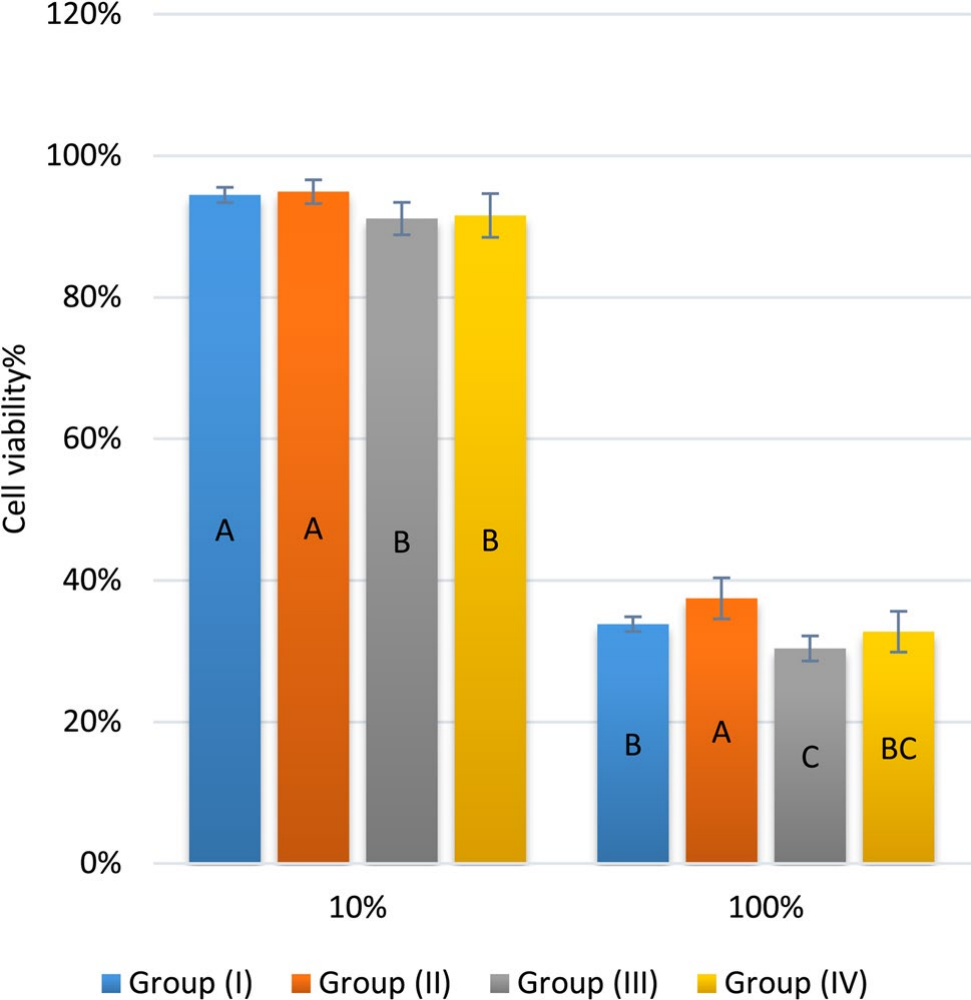
Bar chart displaying the mean and SD values of cell viability (%). (Different superscripts indicate a statistically significant difference at the same concentration)

The means and SD of cell viability (%) are presented in Table 2; Fig. 4. The differences between the mean values of all groups were statistically significant. At the 10% concentration, the mean cell viability (%) values of groups I and II were statistically significant higher than those of the other groups (*p* = 0.007). Meanwhile, at the 100% concentration, the mean cell viability (%) value of group II was statistically significantly higher than that of the other groups (*p* < 0.001). In addition, group I had a statistically significantly higher mean value of cell viability (%) than did group III (*p* < 0.001). For all groups, the extracts at the 10% concentration had significantly higher mean values than those at the 100% concentration (*p* < 0.001).

**Table 2:** Simple effects comparisons and summary statistics of cell viability (%)

Conc.	Cell viability (%) (Mean ± SD)				*p*-value
	Group (I)	Group (II)	Group (III)	Group (IV)	
**10%**	94.47 ± 1.08^A^	94.92 ± 1.68^A^	91.12 ± 2.29^B^	91.56 ± 3.09^B^	**0.007** *
**100%**	33.81 ± 1.04^B^	37.46 ± 2.90^A^	30.38 ± 1.77^C^	32.74 ± 2.88^BC^	**< ** **0.001** *
***p*** **-value**	**< ** **0.001** *	**< ** **0.001** *	**< ** **0.001** *	**< ** **0.001** *	

Values with different superscripts within the same horizontal row are significantly different

* Significant (*p* < 0.05)

### Compressive strength test

**Fig. 5:**
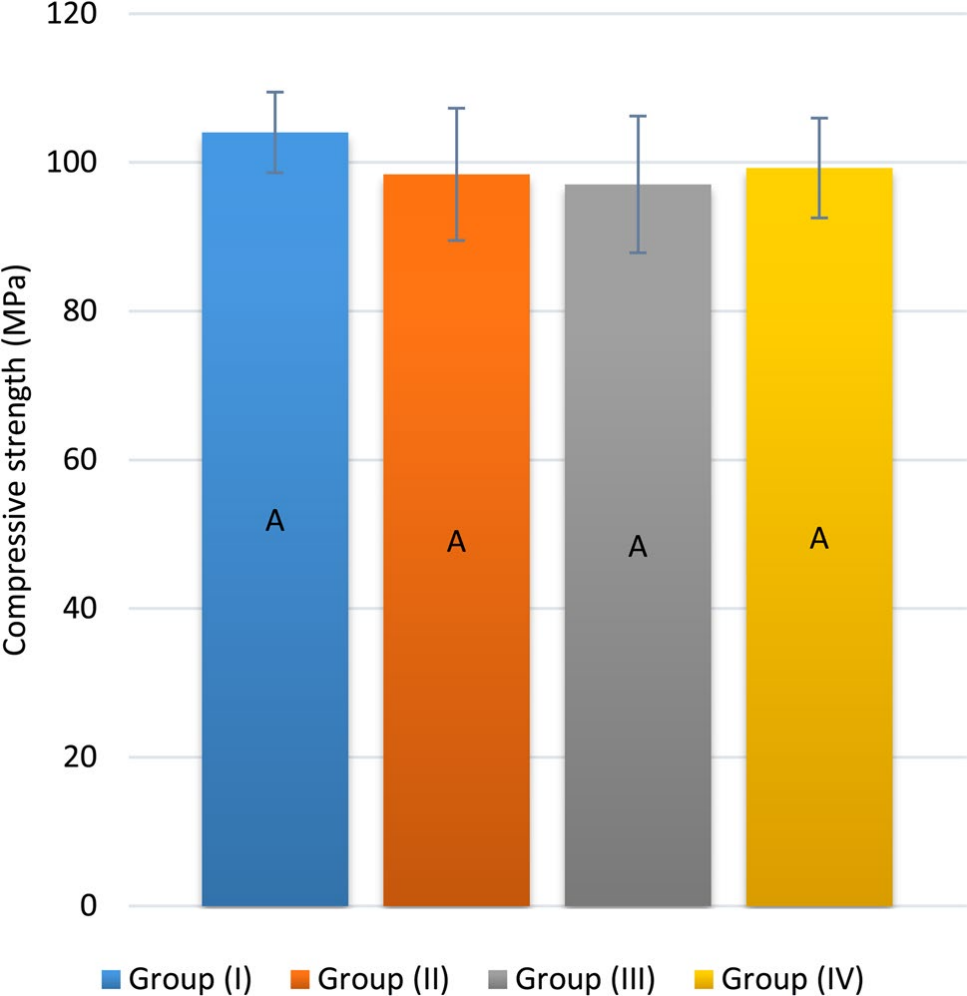
Bar chart displaying the mean and SD values of compressive strength (MPa). (Different superscripts indicate a significant difference)

The mean values and SD of the compressive strength (MPa) are presented in Table 3; Fig. 5. The mean values of all groups did not differ significantly from one another (*p* = 0.614).

**Table 3:** Intergroup comparison and summary statistics of the compressive strength (MPa)

Compressive strength (MPa) (Mean ± SD)				*p*-value
Group (I)	Group (II)	Group (III)	Group (IV)	
104.04 ± 5.43^A^	98.40 ± 8.90^A^	97.05 ± 9.19^A^	99.26 ± 6.71^A^	**0.614ns**

*ns* not significant

Values with different superscripts are significantly different

## Discussion

This study demonstrated that chemically synthesized NSe has an antibacterial effect against *S. mutans*, with a MIC value of 37.5 ppm. In addition, NSe-modified GIC showed extended antibacterial effects against *S. mutans* for up to 7 days, without a statistically significant reduction in cell viability or compressive strength. Based on these results, the null hypothesis was rejected for antibacterial activity, while it was accepted for cytocompatibility and compressive strength.

Dental caries is considered the most prevalent chronic oral disease according to the World Health Organization (WHO) [33]. *S. mutans* is the bacterium most commonly associated with dental caries. These gram-positive cocci are highly acidogenic and can metabolize a wide range of carbohydrates. Furthermore, they possess specific cell surface proteins that assist in primary adhesion to the tooth and facilitate bacterial colonization and biofilm formation [7–9, 21]. Antibacterial activity against *S. mutans* is the principal property to be analyzed when proposing a restorative material with antibacterial features [34]. The direct contact killing effect on bacteria could be the main reason for early-stage biofilm inhibition, as biofilm-inhibiting properties result from a combined effect of antibacterial material release and surface contact killing on the bacteria [35]. For these reasons, in this study, *S. mutans* was chosen for MIC determination and antibacterial activity evaluation.

MIC was measured via the broth microdilution technique, which has advantages over the macrodilution method, including reproducibility, a lower risk of errors, and economy of substances and space [36]. MIC was found to be equivalent to 37.5 ppm, in accordance with the results of Yazhiniprabha and Vaseehara [37]. Unlike the value reported by Darroudi et al. [21], the MIC was 68 µg/mL, as a result of using a different strain of *S. mutans* (PTCC No: 1683).

In our study, GIC was modified with three different concentrations of NSe suspensions (75, 112.5, and 150 ppm). These concentrations were determined by doubling, tripling, and quadrupling the obtained MIC value (37.5 ppm), respectively. The use of such NSe concentrations is a well-balanced strategy to enhance the antibacterial capabilities of GIC while preserving its biological and physicomechanical features. Notably, the GIC setting time was prolonged as a result of increasing NSe concentration, according to a pilot study performed previously, in which the setting time was evaluated according to ISO 9917-1 [29]. This prolongation upon modification is a frequent occurrence that is thought to result from a potentially impaired interaction between polyacrylic acid and fluorocalcium aluminosilicate glass [1]. A possible explanation is that Se might interact with released calcium or aluminum ions to create new compounds. These interactions may therefore cause the primary setting reactions to be delayed, which would lengthen the setting time. Meanwhile, using concentrations higher than the MIC value was necessary to obtain a GIC with effective antibacterial action against *S. mutans*.

In this study, the NSe-modified groups showed significant antibacterial activity against *S. mutans* ATCC 25175 in a concentration-dependent manner. Group V had statistically significantly higher mean values at all time intervals, followed by group III, which had statistically significantly higher mean values than groups I and II, except at the 7 days interval. The antibacterial activity may be attributed to NSe attaching to the surface of bacterial cells and penetrating through cell walls, causing their disruption [13, 20, 22, 37]. Another suggested mechanism includes the release of Se ions and their interaction with the functional groups of proteins and enzymes, such as -SH or –NH, leading to the loss of their structure and functionality [19]. Additionally, NSe may deactivate normal processes that allow the membrane transport of ions and nutrients through cell walls, thereby preventing essential cell activities [38]. Furthermore, NSe may have photocatalytic effects on bacteria [39]. Moreover, the morphological alterations which might be caused by NSe could disrupt respiratory processes and ATP synthesis. This would prevent cell division and cause membrane depolarization and cell integrity degradation, ultimately leading to microbial cell death [12].

Initially, NSe might adhere to bacteria to act on them. This depends on the molecular components and charges of both NSe and bacterial cell walls [13]. Positively charged NSe could elicit enhanced antibacterial activity against negatively charged bacterial cell walls. In gram-positive bacteria, the cell wall is thick and consists mainly of multiple peptidoglycan layers without an outer lipopolysaccharide membrane, and the net surface charge is negative [13, 20, 21]. NSe might be readily deposited through electrostatic interactions in the peptidoglycan layer of gram-positive bacteria, thereby disrupting bacterial cell division [21].

The increase in antibacterial activity with increasing NSe concentration may be attributed to the fact that higher concentrations increase the area of contact, which enhances the antibacterial effects [40]. In addition to breaking down the cell walls, increased Se ion concentrations would also compromise the integrity of the cell membrane, altering intracellular homeostasis and ultimately resulting in microbial cell death. This is crucial, since the cell wall and membrane defenses are the origins of microbial resistance [11]. These findings are consistent with those of Hou et al. [40], Hamman et al. [41], and Geoffrion et al. [23].

The NSe-modified GIC showed a persistent antibacterial effect, which extended for up to 7 days at relatively high NSe concentrations, with group V having a mean IZO 1.21 mm larger than that of group I. This could be explained by the formation of Se compounds due to the interaction of Se with the GIC constituents. These newly formed compounds could have released Se ions over time, causing the continued antibacterial effect. Another theory suggests that some NSe may have been incorporated into the voids of the cement during mixing [1], thereby contributing to its eventual release.

The incorporation of antibacterial agents into restorative materials frequently interferes with their biocompatibility and compressive strength [1, 6]. Antibacterial agents that rely on ROS generation to kill microorganisms are of great concern. It is commonly recognized that ROS are double-edged swords that, in addition to killing microbes, may damage various human cells [18].

The SRB assay is a fluorescent colorimetric assay for determining cell density and is a popular and dependable technique with superior predictive power over the 3-(4,5-dimethylthiazol-2-yl)−2,5-diphenyl-2 H-tetrazolium bromide (MTT) assay recommended by ISO [42, 43]. A previous study by Van Tonder et al. [44] reported that the SRB assay is the only true cell enumeration method compared to the MTT assay because it does not depend on the metabolic activity of living cells. Rather, it depends on the capacity of the SRB stain to attach to the basic amino acids of cell proteins in a mildly acidic environment, releasing the stain when the environment becomes strongly basic. This removes the impact of fluctuating biological factors on the quantification process. According to these data, the SRB assay works best for identifying both minor and major variations in the number of cells [44].

The potential applications of NSe rely precisely on its selective cytotoxicity, meaning that NSe exhibits effective cytotoxicity against bacteria while remaining safe for eukaryotic cells. Numerous earlier investigations have demonstrated that NSe has mild cytotoxic effects along with outstanding antibacterial activity [12, 23, 37, 40, 45], and that its cytotoxicity is both time- and concentration-dependent [12, 40, 45]. Moreover, at low concentrations, NSe can enhance the viability of MC3T3-E1 osteoblasts [40, 46] and cerebral cortex cells [47].

Given its dual nature, Se acts as a pro-oxidant at supernutritional levels and as an antioxidant at subnutritional levels [11]. In this study, the viability of oral epithelial cells increased with the use of NSe at low concentrations, such as 75 ppm. The lack of NSe toxicity at low concentrations can be explained by its antioxidant properties through the suppression of H_2_O_2_ and ROS production [10, 12, 37, 47, 48], as oxidation produces free radicals that initiate chain reactions and damage the cells [10]. Under both in vitro and in vivo conditions, Se compounds, including methylselenocysteine, selenomethionine, selenocystine, and Se amino acids, can be very effective at scavenging free radicals and halting the oxidative degradation of DNA [10, 14]. According to Varlamova et al. [47], NSe can inhibit ROS production during hypoxia and ischemia/reoxygenation. Additionally, it can promote cell survival in the penumbra zone by activating mitochondrial biogenesis and preserving intracellular ATP and Ca^+ 2^ homeostasis at normal levels. Nevertheless, Xia et al. [48] reported that NSe stabilized with Cts has substantial immunomodulatory activity. Lipid metabolism and antioxidant activity are closely linked to this immunomodulatory effect.

Another assumption is that NSe may interact with the calcium and aluminum ions released from GIC to create new compounds. This interaction eliminates the cytotoxic effect of aluminum ions [49, 50]. Shehab et al.’s work [51], which involved adding NSe to mineral trioxide aggregate, served as the basis for this argument. After setting in the altered samples, a novel chemical known as “calcium selenite” was identified. Additionally, the ability of Se to interact with aluminum has been validated in several studies [49, 50]. Based on this affinity, Ghorbel et al. [49] and Cao et al. [50] suggested the use of Se to prevent aluminum-induced toxicity.

The dental material should be strong enough to withstand various stresses. Compressive strength is the most important measure to investigate because most masticatory forces are compressive in nature [2, 5, 52]. A compressive strength test was performed after the samples were stored for 24 h to ensure that final setting was achieved [53]. No statistically significant difference was detected between any of the groups. This finding was in line with the results of El-Wassefy et al. [52], Jessy et al. [6], and Guo et al. [7], where the addition of an antibacterial agent did not significantly affect the GIC compressive strength.

Unlike Yan et al. [54] and Bayoumi and Habib [27], the addition of antibacterial agents significantly altered the compressive strength of GIC, as it decreased. The compressive strength is significantly affected by the nature and concentration of antibacterial agents. An increased NPs concentration may hinder the maturation process and disrupt the continuity of the GIC matrix and its cross linking, which would reduce the cohesive strength between the material particles [27, 53].

Meanwhile, for Kashyap et al. [55], Arafa et al. [3], Ge et al. [2], and Hamdy [5], the compressive strength of the antibacterially altered GIC significantly increased. This might have occurred since, during the mixing procedure, these tiny NPs were incorporated into the voids of the GIC [1]. Moreover, the use of very small amounts of nanofillers may prevent crack propagation by shifting stress from the weaker matrix to the stronger NPs [3, 5]. Nevertheless, adding NPs to GIC has advantages only up to a limit, after which the strength characteristics of GIC begin to deteriorate [1].

The modification of GIC with NSe resulted in extended antibacterial effects against *S. mutans* for up to 7 days without significantly compromising cell viability or compressive strength, resulting in a promising restorative material for ART. Moreover, the viability of human oral epithelial cells was increased by altering the GIC with NSe at low concentrations, such as 75 ppm, which may be beneficial for treating class V cavities. However, there are some limitations to note. This study is an in vitro investigation that does not simulate the complex environment of the oral cavity; in vivo testing is essential for evaluating clinical performance. The solubility and release kinetics of Se and other ions were not studied. The evaluation of antibacterial activity against multi-strains of bacteria and biofilm formation on modified GIC should be considered in further studies. Furthermore, long-term evaluation needs to be explored in future research.

## Conclusions

Within the limitations of the current study, our findings suggest that NSe-modified GIC, up to 150 ppm, may offer improved antibacterial performance without compromising safety, and may present a promising restorative material for ART. Meanwhile, the improvement in cell viability at low concentrations of NSe, such as 75 ppm, may be beneficial for treating class V. Additionally, the modification of GIC with NSe at different concentrations did not compromise its compressive strength.

## Data Availability

The datasets used and/or analyzed during the current study are available from the corresponding author upon reasonable request.

## Abbreviations

GIC: Glass ionomer cement
ART: Atraumatic restorative treatment
Se: Selenium
ROS: Reactive oxygen species
NPs: Nanoparticles
NSe: Nanoselenium
*S. mutans*: *Streptococcus mutans*
Cts: Chitosan
TEM: Transmission electron microscopy
DLS: Dynamic light scattering
ZP: Zeta potential
MIC: Minimum inhibitory concentration
BHI: Brain heart infusion
IZO: Inhibition zone
SRB: Sulforhodamine B
SD: Standard deviation
MTT: 3-(4,5-dimethylthiazol-2-yl)-2,5-diphenyl-2H-tetrazolium bromide
